# Supplementary material for: Precise 3D Tracking of Highly Non‐Planar Eukaryotic Flagellar Beating Patterns Using Digital Holographic Microscopy
Source: Small Methods. 2026 May 6;10(12):e01589. doi: 10.1002/smtd.202501589 (PMC13288012; doi:10.1002/smtd.202501589)
Supplement: Supplementary file 1 — Supporting File 1:: smtd70682‐sup‐0001‐SuppMat.docx. [file SMTD-10-e01589-s002.docx]

Supporting Information

Precise 3D Tracking of Highly Non-planar Eukaryotic Flagellar Beating Patterns using Digital Holographic Microscopy

Patryk Nienaltowski, Jonasz Slomka, Federica Miano, Thomas Kiørboe, Clara Martínez-Pérez, Tristan Colomb, Yves Emery*, and Roman Stocker*

This Supporting Information file includes:

- Figures S1 to S2
- Legend for Movies S1 to S2
- Legend for Data S1 to S6


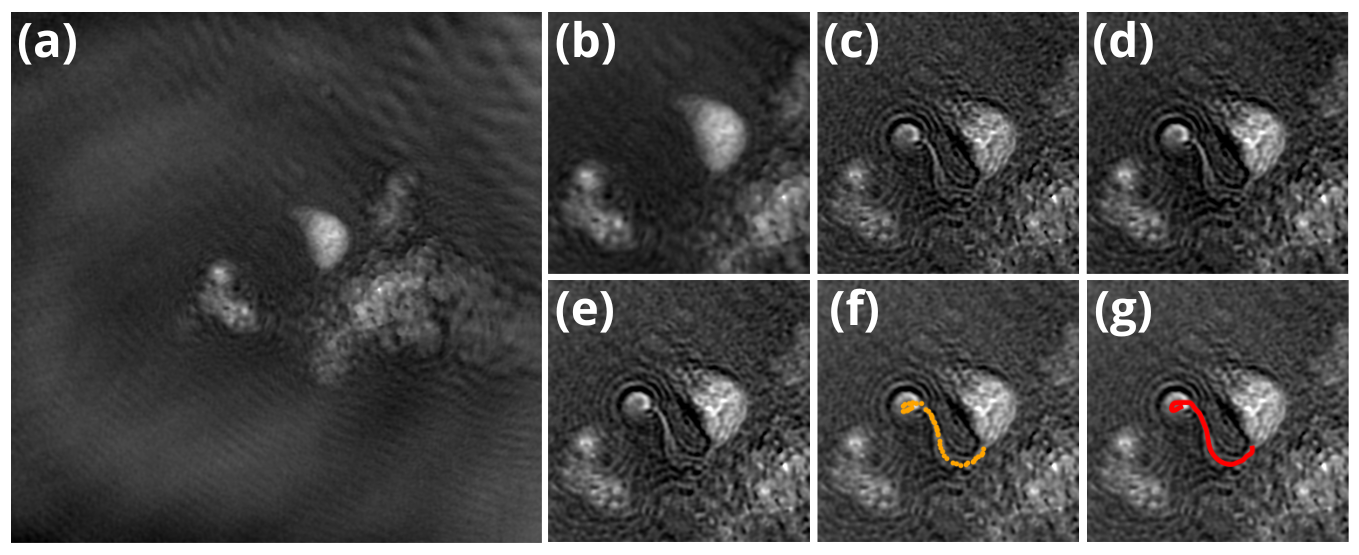


**Figure S1**. Image processing and segmentation of a *R. americana* flagellum in the xy plane. (a) Phase reconstruction at a single *z* plane. (b) Region of interest (ROI) centered on the cell, expanded to capture the full extent of the flagellum. (c) 3D temporal filtering suppresses background, noise, and stationary features, enhancing flagellum visibility. (d) Gaussian filtering further reduces high-frequency noise. (e) Contrast enhancement by histogram stretching using percentile-based limits (vmin, vmax = 0.5th and 99.95th percentiles). (f) User-defined lateral positions along the visible anterior flagellum (orange dots). (g) Smoothed flagellum shape (red).


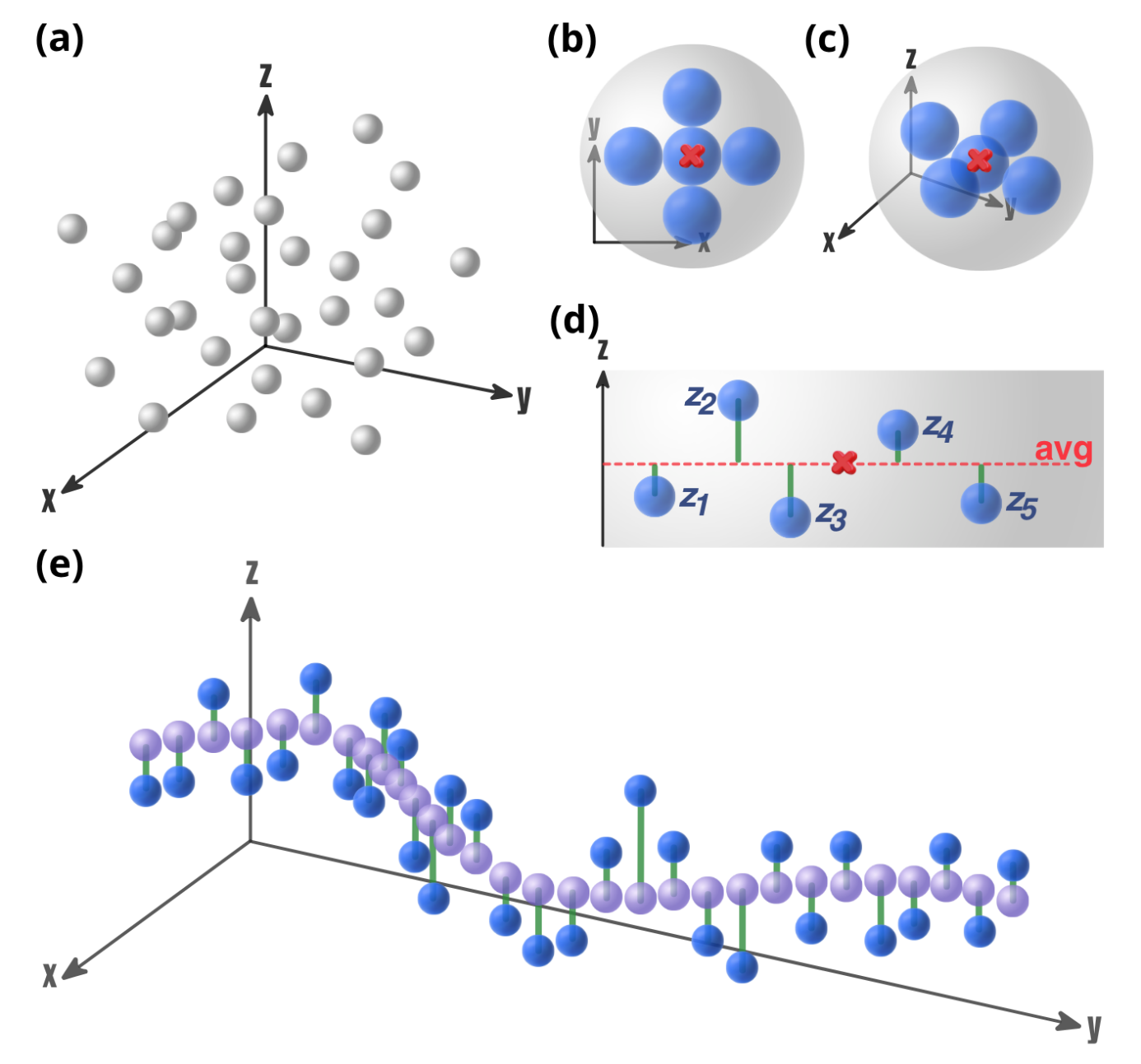


**Figure S2**. Axial resolution assessment workflow. (a) 3D distribution of randomly dispersed polystyrene particles within the measurement volume. (b) Definition of lateral sampling positions within a single particle using five points arranged in a Von Neumann neighborhood, consisting of one central point and four peripheral points. The corresponding reference axial position, defined as the mean of the axial positions of these points, is indicated by a red cross. (c) 3D representation of the same particle showing the axial positions calculated for each of the five points. The reference axial position, defined as the mean of all five axial positions within the particle, is indicated by a red cross. (d) Definition of axial residual error as the difference between the axial position of each point and the corresponding reference axial position (e) Extension of the procedure to a motile flagellum, where axial residual errors are computed for each tracked point relative to a smoothed flagellar centerline, obtained by filtering the axial positions along the flagellum.

# Movie S1.

Three-dimensional tracking of the mouse spermatozoa flagellum. The left clip shows a two-dimensional phase reconstruction on a single *z*-plane after 3D temporal filtering, with lateral localization of the anterior flagellum highlighted in red. Center and right clips show the flagellum’s 3D beating pattern at distinct elevation and azimuth angles to illustrate its full kinematics. Video is slowed 2× to enhance visualization of the flagellar beat.

# Movie S2.

Three-dimensional tracking of the *R. americana* flagellum. The left clip shows a two-dimensional phase reconstruction on a single *z*-plane after 3D temporal filtering, with lateral localization of the anterior flagellum highlighted in red. Center and right clips show the flagellum’s 3D beating pattern at distinct elevation and azimuth angles to illustrate its full kinematics. Video is slowed 40× to enhance visualization of the flagellar beat.

# Data S1. (separate file)

Results of 3D tracking of mouse spermatozoa flagellum. Processed (*x*,*y*,*z*) coordinates for each frame of the analyzed sequence are provided in individual worksheets, named by frame number. The “metadata” worksheet details the experimental settings. The “all_data” worksheet concatenates the full time series of 3D coordinates for the analyzed sequence.

# Data S2. (separate file)

Results of 3D tracking of *R. americana* flagellum. Processed (*x*,*y*,*z*) coordinates for each frame of the analyzed sequence are provided in individual worksheets, named by frame number. The “metadata” worksheet details the experimental settings. The “all_data“ worksheet concatenates the full time series of 3D coordinates for the analyzed sequence.

# Data S3. (separate file)

Processing time logs for lateral and axial localization in the mouse spermatozoa flagellum 3D tracking pipeline. For each frame, the elapsed times (s) of each core stage of the tracking pipeline are recorded. The worksheet summarizes average and standard deviation for each stage across the full sequence. The “metadata” worksheet details the experimental settings.

# Data S4. (separate file)

Three-dimensional coordinates of the mouse spermatozoa flagellum used for the axial resolution analysis. Processed (*x*,*y*,*z*) coordinates for each frame of the analyzed sequence are provided in individual worksheets, named by frame number. The “metadata” worksheet details the experimental settings.

# Data S5. (separate file)

Three-dimensional coordinates of the dispersed polystyrene 1.05-μm research particles used for the axial resolution analysis. Processed (*x*,*y*,*z*) coordinates for each frame of the analyzed sequence are provided in individual worksheets, named by frame number. The “metadata” worksheet details the experimental settings.

# Data S6. (separate file)

Axial resolution metrics. Consolidated performance table summarizing root-mean-square error (RMSE), mean bias error (MBE), and maximum error in axial localization for both test objects - mouse spermatozoa flagellum (Data S4) and polystyrene particles (Data S5). All metrics are reported in micrometers. The “metadata” worksheet details the experimental settings.
